# Combined pulmonary lobectomy for surgical treatment of a malignant fibrous histiocytoma of the chest wall: a case report

**DOI:** 10.1186/1746-1596-9-21

**Published:** 2014-01-23

**Authors:** Yi Liu, Gang Chen, Yi Wu, Renwang Liu, Song Xu, Jun Chen, Qinghua Zhou

**Affiliations:** 1 Department of Lung Cancer Surgery, Tianjin Lung Cancer Institute, Tianjin Medical University General Hospital, Tianjin 30052, China

**Keywords:** Malignant fibrous histiocytoma, Differential diagnosis, Chest wall tumor

## Abstract

**Background:**

A malignant fibrous histiocytoma (MFH) rarely originates from the chest wall.

**Clinical findings:**

In this case, we describe a 59-year-old Chinese woman who presented with an enormous mass originating from the left chest wall and involving the left upper pulmonary lobe.

**Therapy:**

After a radical en-block resection of the entire chest mass with left upper pulmonary lobectomy, and the chest wall reconstruction, a histopathologic diagnosis of the giant cell MFH was rendered. She has done well postoperatively, showing no local recurrence or distal disease in an 8-month follow-up period.

**Conclusion:**

Although a MFH originating from the chest wall is rare, it should be considered in the differential diagnosis of a chest wall tumor.

**Virtual slides:**

The virtual slide(s) for this article can be found here: http://www.diagnosticpathology.diagnomx.eu/vs/8895569301129379

## Introduction

Malignant fibrous histiocytoma (MFH) is a common soft tissue sarcoma, which is often found in adults with primary location of the extremities or retroperitoneum. However, MFH rarely originates from the chest wall [1]. Here we report the case in a 59-year old woman with a left chest big mass involving the left upper pulmonary lobe that was initially suspicious of lung carcinoma invading the chest wall. Ultimately, the chest wall giant cell malignant fibrous histiocytoma was confirmed following en-block resection of chest wall mass and left upper lobectomy.

## Case presentation

A 59-year-old Chinese woman was admitted to the hospital with the chief complaint of a progressively enlarging mass in the left thoracic wall over the past seven months. At the initial examination, the tumor slightly protruded from the chest wall with the 2 cm in diameter, and gradually grew over in the past several months. In one month ago, she underwent a percutaneous transthoracic needle biopsy of the mass in another hospital. Following the puncture, the tumor promptly increased significantly in size, impacting blood flow to the left arm. On this admission, we conducted a physical examination and palpated the soft painless left lateral chest wall large mass with the 9 cm in diameter. She denied smoking history and had no family history of lung cancer. A review of her systems was noncontributory. The results of a peripheral blood count, baseline serum chemistry screening, and urinalysis were normal on admission, as were tumor biomarker tests and a purified protein derivative test for tuberculosis.

**Figure 1:**
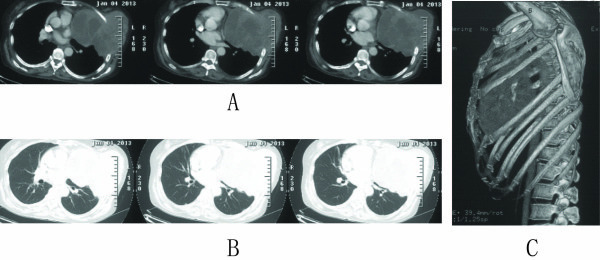
**Enhanced chest CT scan before operation. A**: In the mediastinal window, the CT scan revealed the tumor encroaching on the superior vena cava (right panel), surrounding the right upper lobe bronchus (middle panel), and invading the right pulmonary artery (left panel). **B**: In the lung window. **C**: In the mediastinal window, coronal.

Enhanced chest computed tomography (CT) with three-dimensional reconstruction of the ribs showed an enormous soft tissue mass in the left chest wall (Figure 1), which extended into the left lung and mediastinum, exerted pressure on the heart, skewing it to the right, and had eroded the middle portion of the fifth rib. The density of the tissue mass was uneven and a necrotic area was visible. The maximum cross-sectional diameter of the tumor mass was 13.3 × 15.7 cm^2^. Moreover, an ECT bone scan also demonstrated the destruction of the middle of the fifth rib. In additional, a CT scan of her abdomen and magnetic resonance imaging of her brain were all normal. Her bronchial tree also appeared normal on bronchoscopic examination, with no indication of malignancy in the biopsy and washings procured.

After anesthesia and a double-lumen intubation had been established, a left posterolateral incision was performed. The chest wall tumor invaded the left upper pulmonary lobe as well as the fourth, fifth, and sixth ribs. The tumor had eroded the middle portion of the fifth rib; the heart was impacted by the tumor and mediastinal pleural adhesions were present. We freed-up the outer margin of the tumor and excised the fourth, fifth, and sixth ribs. Intercostal tissue was included with a margin over 5 cm. We extracted the tumor and the invaded left upper lobe of lung to remove the pressure on the heart. We then performed the left upper pulmonary lobectomy. Finally, we joined four 10 × 10 cm^2^ polyester surgical patches (knitted type) into one 20 × 20 cm^2^ patch and this patch was sutured to the chest wall defect for chest wall reconstruction.

**Figure 2:**
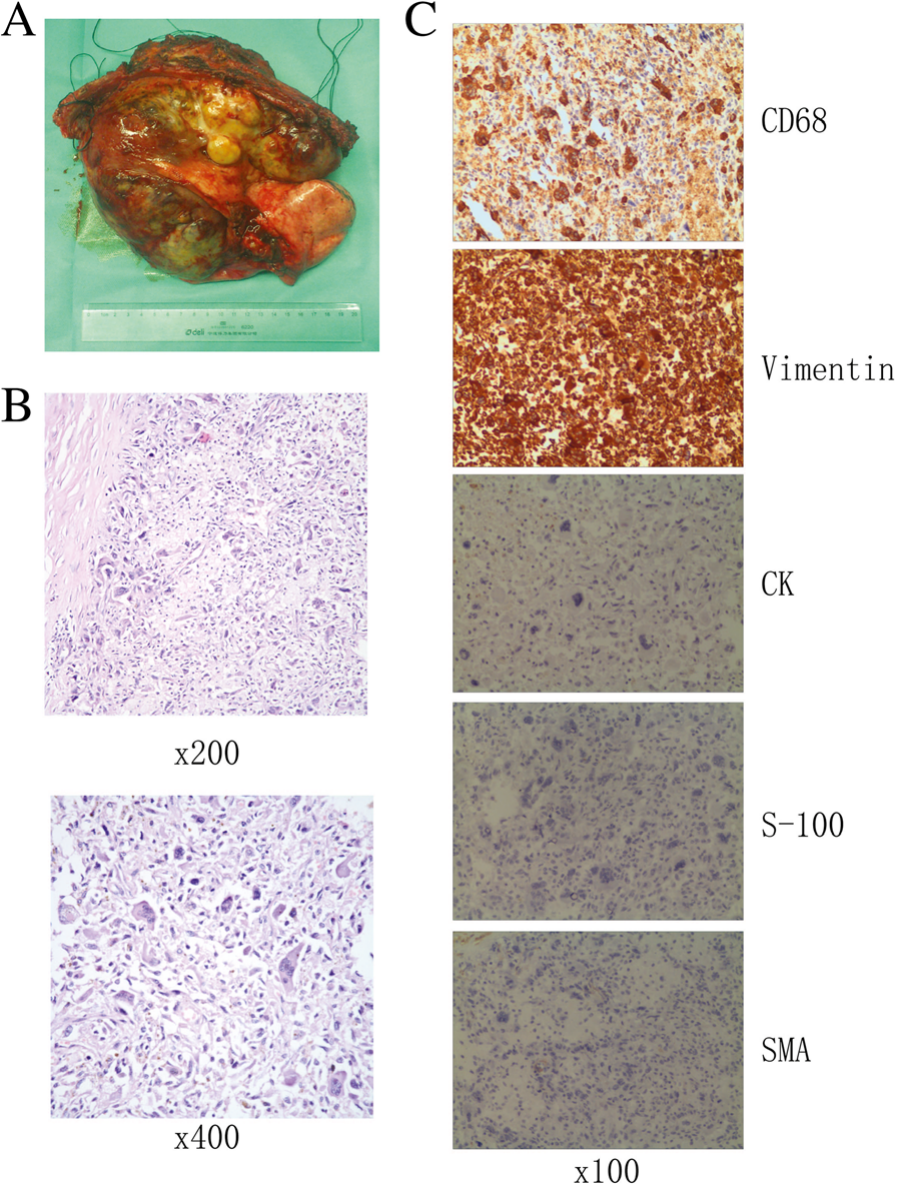
**Histopathological images. (A)** Gross features of the 20 cm mass with the invaded fifth rib and the left upper pulmonary lobe; **(B)** Hematoxylin and eosin (H&E) staining of a giant cell malignant fibrous histiocytoma cell tumor. **(C)** Immunohistochemical staining of primary tumor with antibodies to CD68 (+), Vimentin (+), CK (-), S-100 (-) and SMA (-).

**Figure 3:**
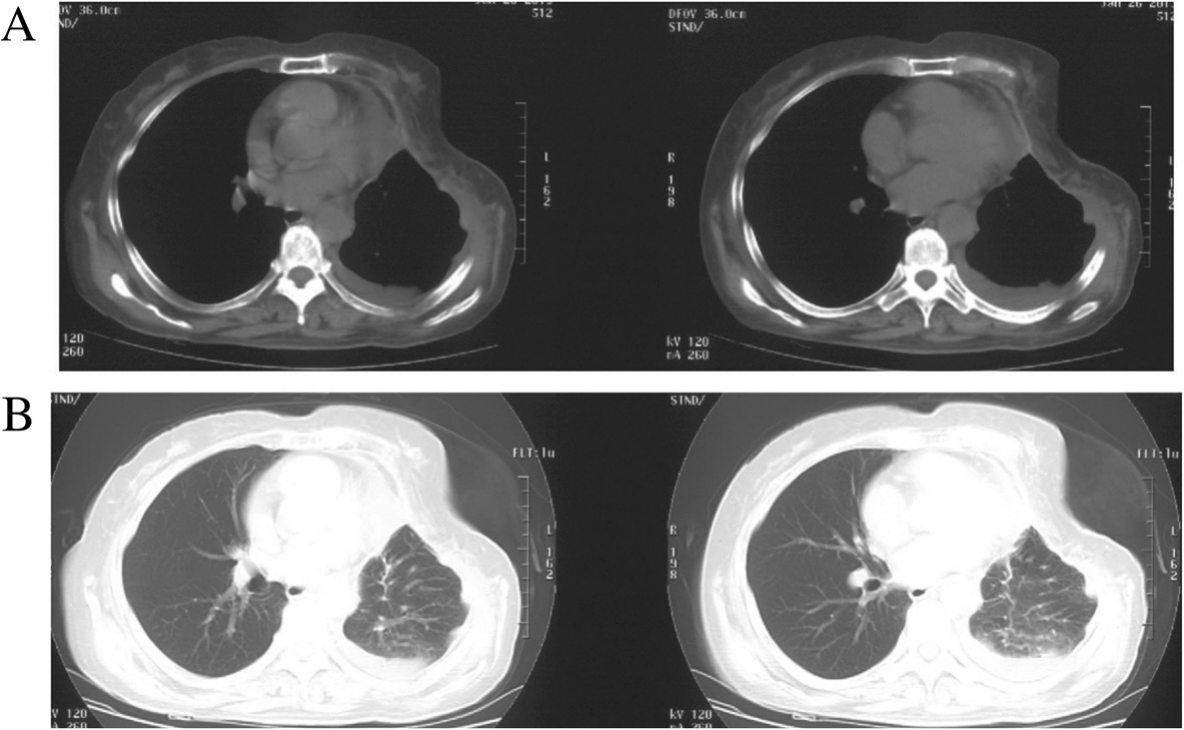
**CT scans after operation.** The chest CT scan was performed in 7 days after the operation. **A**: In the mediastinal window; **B**: In the lung window.

The neoplasm, which was removed by surgical resection, weighed 2.080 kg and was 20 cm × 18 cm × 9 cm in size, as shown in Figure 2A. There were multiple nodules on its surface and a cross section of the tumor was honeycombed in appearance. The pathology report described a giant cell malignant fibrous histiocytoma cell tumor (Figure 2B), with the immunohistochemistry of CD68 (+), Vimentin (+), SMA, CK and S-100 (-) (Figure 2C), invading the left upper lung lobe and the middle of the fourth, fifth, sixth ribs, with no metastases to the mediastinal lymph nodes. Her postoperative course was uneventful. She was discharged 12 days after surgery and showed no signs of local recurrence or distal disease at an 8-month follow-up visit (Figure 3).

## Discussion

A MFH is the most common soft tissue tumor found in adults. It is primarily located in the extremities or retroperitoneum; however, it rarely appears on the chest wall. A MFH was first reported by O’Brien and Stout in 1964 [2]. They described it as “malignant fibrous xanthoma”. The tumor preferentially involves the deep fascia, skeletal muscles, or superficial subcutaneous tissue. A MFH has a high propensity for local recurrence and distant metastases. Weiss and Enzinger [3] reported that a local recurrence rate of 44% and metastatic recurrence rate of 42% for MFHs. Several MFH subtypes have been described in the literature: storiform-pleomorphic, giant cell, inflammatory, and myxoid [4]. The mean survival of patients without surgery is 11.7 months; it is 23.2 months for patients who undergo surgery [5]. MFHs have been reported to occur with increasing frequency in patients treated with radiotherapy for other malignant diseases; however, no prior exposure to ionizing radiation or history of malignancy was documented in our case. The role of either chemotherapy or radiotherapy as primary or adjuvant treatment for MFHs is currently unclear. Favorable factors for MFHs are: UICC/AJCC stage I and II, superficial location, myxoid type, and patient under 50 years of age. Under these conditions, the rational for radical en-block resection of the tumor is supported [6-10].

CT and magnetic resonance imaging are useful for the radiological evaluation of the soft tissue component. According to one report, the imaging features of MFHs in the chest wall are nonspecific and only the myxoid type showed a high-intensity pattern with T1 weighed images (T1WI) and T2 weighted images (T2WI). The mass usually shows intense enhancement on CT with a clear margin separating it from the surrounding tissue [11].

For a chest wall MFH, wide resection is the first treatment choice; chemotherapy should also be considered. King reported the relationship between the distance of the lateral margin of the excision from the tumor and the recurrence rate. They found a distance of 4 cm was associated with a recurrence rate of 44%; whereas a distance of 2 cm was associated with a 71% recurrence rate [12]. They recommended an excisional distance of 4 cm. Naoya Yoshida et al. reviewed 39 MFH cases and found that patients with a negative surgical margin were alive without recurrence, irrespective of the surgical margin distance [12].

After radical en-block resection, the chest wall should be reconstructed. Classically, various flaps such as a major pectoral muscle flap, a major pectoral myocutaneous flap, or a latissimus dorsi myocutaneous flap and pedicled omentum have been utilized for reconstruction. Currently, reconstruction with Marlex mesh is widely used. Several studies have reported that a chest wall reconstruction with Marlex mesh is successful and inexpensive. In this case, the chest wall defect was too large for reconstruction with a flap. We performed the reconstruction with a polyester surgical patch (knitted type), and we joined four 10 × 10 cm^2^ patches into one 20 × 20 cm^2^ patch to reconstruct the chest wall. Postoperative complications such as skin dehiscence, skin necrosis, and infection did not occur. We found that reconstruction of the chest wall with a polyester surgical patch for a large defect is safe, rapid, and simple. We consider that radical en-block resection for an enormous chest wall MFH and reconstruction is a safe procedure that may increase long-term survival.

In differential diagnosis of a chest wall tumor, malignant fibrous histiocytoma should be seriousely considered.

## Conclusion

Malignant fibrous histiocytoma be considered in the differential diagnosis of a chest wall tumor.

## Ethical approval

Our research has been approved by the ethical committee of Tianjin Medical University General Hospital.

## Consent

The patient granted written informed consent for publication of this manuscript and the accompanying images. A copy of the written consent is available for review by the Editor-in-Chief of this journal.

## Competing interests

The authors declare that they have no competing interests.

## Authors’ contributions

YL collected all data and authored the manuscript. GC, YW, RWL and SX were responsible for patient care and analysis of follow-up data. QZ participated in data analysis and manuscript revisions. JC and QHZ performed the surgical procedure, also contributing to data analysis and shaping of the manuscript. All authors have read and approved the final manuscript.
